# Dynamic Response of PVDF Cantilever Due to Droplet Impact Using an Electromechanical Model

**DOI:** 10.3390/s20205764

**Published:** 2020-10-12

**Authors:** Guannan Hao, Xiangwei Dong, Zengliang Li, Xiaoxiao Liu

**Affiliations:** 1 College of Mechanical and Electronic Engineering, China University of Petroleum (East China), 66 Changjiang Rd, Huangdao District, Qingdao 266580, China; b18040006@s.upc.edu.cn (G.H.); lizl@upc.edu.cn (Z.L.); z18040061@s.upc.edu.cn (X.L.); 2 College of Electromechanical Engineering, Binzhou University, Binzhou 256600, China

**Keywords:** electromechanical model, droplet-substrate interactions, multi-mode vibration model, droplet splash, PVDF cantilever sensor, surface wettability

## Abstract

The dynamic response of a polyvinylidene fluoride (PVDF) cantilever beam under excitation of water droplet impact is investigated by developing an electromechanical model. In the model, the governing equations of beam motion and output voltage are derived in the theoretical way, such that the voltage across the PVDF layer and the cantilever deflection can be predicted. The motion of the beam is described by the multi-mode vibration model through which more accurate results can be obtained. The predicted results of the model are validated by the experiment. Combined with the experiment and the model, the effect of surface wettability on droplet-substrate interaction mechanisms is investigated, which provides an insight into the improvement of mechanical-to-electrical energy conversion efficiency in raindrop energy harvesting (REH) applications. Results show: (1) the droplet splash on a super-hydrophobic beam surface has a positive effect on voltage generation. The splash limit that affects the reaction force of the impacting droplet is experimentally determined and greatly dominant by the Weber number. (2) Small-scaled droplets in splash regime allow generating higher voltage output from a super-hydrophobic beam surface than from an untreated hydrophilic beam surface. (3) Tests of successive droplet impacts also show that a super-hydrophobic surface performs better over a hydrophilic surface by producing constant peak voltage and higher electrical energy harvested. In this case, the voltage measured from the hydrophilic surface decreases gradually as the water layer is accumulated. Overall, the electromechanical behaviors of a super-hydrophobic PVDF cantilever sensor can be well predicted by the model which shows a great potential in energy harvesting by maximizing the inelastic collision upon droplet-substrate interactions.

## 1. Introduction

As the raindrop energy harvesting (REH) applications are being extensively concerned, harvesters based on piezoelectric beams have shown great potential in energy scavenging. The moderate kinetic energy of raindrops is promising in powering low-energy electronic devices such as remote sensors and piezo-MEMS. Since the first model developed by Guigon et al. [1,2] based on the shock theory combined with momentum conservation, droplet impact on piezoelectric beams is considered as an inelastic collision. The analog made between a piezoelectric material and a capacitor allows to estimate the electrical energy collected and the power output [3]. The current mechanical-to-electrical energy conversion efficiency is on order of 0.1% which still needs further improvement [4]. Hence, efforts have been made in developing novel harvesting structures [5,6,7,8,9,10,11], improving the performance of different piezoelectric materials [12,13,14] and the dynamic outputs from a piezoelectric beam subjected to droplet impact [15,16,17,18,19].

Between the most widely used piezoelectric materials of PVDF (e.g., Polyvinylidene fluoride) and PZT (e.g., Lead Zirconate Titanate), D. Vatansever et al. [12] first demonstrated the ability of PVDF in generating higher voltage output than PZT by performing experiments with various beam dimensions. Further, Viola et al. [20] tested both PZT and PVDF piezoelectric beams and showed that the maximized output power was achieved with a PVDF beam of 25~30 mm in length and 3.3 mm in width. Compared to the brittle nature of PZT which can cause fatigue failure under high frequency cyclic load especially in outdoor utilizations, the PVDF performs better due to its high flexibility, lightweight, low acoustic and mechanical impedance. Therefore, the PVDF is being increasingly used and many available commercial PVDF sensors also provide convenience for conducting impact tests. However, only a prototype of energy harvesters made by a PZT bimorph cantilever was developed and tested in both simulated and actual rain conditions [21,22]. For PVDF harvesters, the electromechanical behaviors as well its superiority to PZT harvesters still need to be further explored before being put into service.

As the basis of the typical prototypes of harvester, cantilever-style piezoelectric sensors have been extensively studied theoretically and experimentally. Wong et al. [23] compared the electric output from bridge and cantilever piezoelectric beams where both open-circuit voltage and closed-circuit voltage were tested, and the mechanical behavior of the beam was analyzed using the first-order vibration mode. Ilyas et al. [15] identified the voltage profile generated from a cantilever-style harvester by a log growth stage and an exponential decay stage. The energy conversion efficiency was generally less than 0.12% but it was suggested that the efficiency can be improved by exploring new surface materials to maximize inelastic collision. For previous studies, only the untreated beam surface of harvesters were used, no new surface has been tested. Also, the droplet dynamic behaviors altered by distinct surface properties of the beam were rarely concerned which may have a no neglected effect on electrical output. In fact, understanding the mechanism of droplet impact dynamics is essential to further optimizing the efficiency of piezoelectric raindrop energy harvesters.

A droplet can generally experience spreading, recoil/rebound and splash phases of impact along with the interchange of kinetic, surface tension and strain energies. Previous researchers [24,25] believed that droplet experiencing higher impact velocities triggers the droplet splash which leads to the mechanical energy loss of the system. The droplet kinetic energy is delivered by a large amount of satellite drops formed at initial stage of impact. It was also suggested that [9,26] the droplet-substrate collision during impact is not complete due to the splash phenomenon which is must associated to an impact efficiency. As the droplet splash is an important impact mechanism that affects the voltage generation hence the energy conversion efficiency, several dimensionless numbers such as Weber number (*We*) and Reynolds number (*Re*) can be employed (seen in Equations (1) and (2)) where *ρ_d_*, *µ* and *γ* is the density, dynamic viscosity and surface tension of water droplet respectively, *V_d_* denotes the impact velocity and *D_d_* represents the droplet diameter. An empirical criterion developed by Mundo et al. [27] (seen in Equation (3)) has been extensively used in determining the splash limit, but neither the substrate flexibility nor the surface property is taken into account. Actually, the splash limit can be altered by the surface wettability characterized by the contact angle. The hydrophilic (H) and super-hydrophobic (SH) surfaces can contribute to the sticking and repelling effect of the droplet respectively. For the former, water layer deposited on the surface may lead to pre-strain of the beam thus less strain energy can be scavenged; whereas droplet on the latter is more prone to splash which leads to mechanical energy loss. Therefore, the effect of surface wettability on dynamic response of the harvester needs to be further investigated which was only qualitatively described in literatures.

(1) $$We=\rho_{d}V_{d}^{2}D_{d}/\gamma ,$$

(2) $$Re=\rho_{d}V_{d}D_{d}/\mu ,$$

(3) $$Re^{0.25}\times We^{0.5}>57.7.$$

The dynamic response of a PVDF cantilever is the core of energy harvesters for which the deformation of the cantilever (i.e., transverse displacement) under excitation of droplet impacts should be first addressed. To model the electromechanical behaviors of the harvester, most previous studies only considered the first-mode vibration of the beam where the lumped parameter approach were usually applied [28]. However, it was demonstrated [29] that the lumped parameter model may yield inaccurate results because it only considers a single viscous damping. A correction factor of the external viscous damping is needed for improving the transverse vibrations. In this case, a distributed parameter model is advantageous which allows to accurately predict the electromechanical behaviors of the beam by means of accurate strain distribution. Based on the model developed by Sodano et al. [30] using Rayleigh–Ritz theorem and Hamilton’s principle, Wong et al. [31] tried to model both the impact force of droplet and the force generated by the water ripple on a PZT cantilever through applying the distributed parameter method. Results showed that the presence of water layer on an untreated beam surface decreased the dominant frequency so as the voltage output. The contribution from the water ripple to voltage generation was very low, thus it was suggested to prevent the accumulation of water layer on the beam surface in design of harvesters. Moreover, following the Euler-Bernoulli assumption model proposed by Inman and ErturkIt [32,33], Doria et al. [6,34] recently developed a multi-mode model for a novel PZT cantilever harvester equipped with a tip spoon. It showed that the sequence of phenomena related to the impact on the liquid surface leads to more energy harvested and the simulated results fitted well with the experiments. But the model was only validated with single droplet of 1mm falling at a speed of 4 m/s, and the flexibility of the model related to impact parameters (i.e., droplet size, impact velocity, etc.) was not concerned. In this study, a multi-mode electromechanical model of PVDF cantilever is developed following the distributed parameter approach. It is validated by experiments for a wide range of droplet sizes (i.e., 2.4 mm~4.6 mm) and impact velocities (i.e., 0.9 m/s~3.4 m/s). The surface wettability effect on voltage output is investigated by fabricating SH beam surfaces. To capture the fundamental physics upon droplet-substrate interactions, a high-speed camera is used. Combined with the electrical output recorded by a digital oscilloscope, the dynamic response of the harvester can be comprehensively studied.

The remainder of this paper is organized as follows. The electromechanical model of a unimorph PVDF cantilever sensor based on Euler-Bernoulli beam theory is proposed in Section 2. The experiment equipment for realizing impact tests is presented in Section 3. In Section 4, the model is validated by comparing with experiments. Effect of surface wettability on dynamic outputs due to different droplet-substrate interaction mechanisms is investigated. The droplet splash is shown to have a positive effect on energy harvesting. Finally, concluding remarks are given in Section 5.

## 2. Modeling of the PVDF Cantilever Sensor

**Figure 1:**
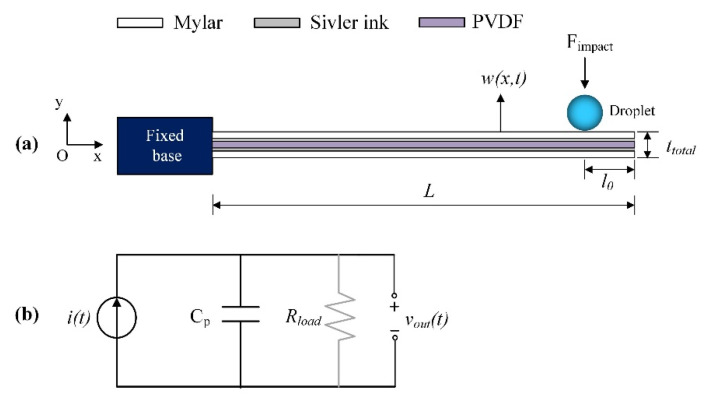
Theoretical model of harvester excited by droplet impact: (**a**) mechanical model of cantilever sensor; (**b**) equivalent electric circuit of PVDF layer.

The commercial PVDF sensor (LDT1-028K) is used as the energy harvesting element (i.e., piezoelectric cantilever beam) in this study. For the sensor, a single PVDF layer is sandwiched between silver inks and protected by exterior layers of Mylar shown as in Figure 1a. The sensor is considered as a uniform composite beam with the clamped-end fixed by the base and the free-end under excitation of droplet impact. The dimension of beam is *L* (length) × *b* (width) × *h* (thickness). The PVDF layer can be represented as a current source *i*(*t*) in parallel with its internal capacitance Cp and connected with external load *R_load_*. The equivalent electrical circuit is shown in Figure 1b. When droplets impinge the beam free-end at a distance *l*_0_ by an impulsive force *F_impact_*, dynamics of the system can be characterized by two aspects: vibration of the cantilever beam and voltage measured across the PVDF layer. Considering multi-mode vibration, the distributed parameter modeling allows for accurate prediction of electromechanical responses including displacement along cantilever length *w*(*x,t*) and the output voltage *v_out_*(*t*) either in an open-circuit condition (without *R_load_*) or in a closed-circuit condition (with *R_load_*).

Following the Euler–Bernoulli assumption, the mechanical equation of motion with electrical coupling can be written in Equation (4) [32] where *YI* is the bending stiffness of the cantilever beam with *I* the equivalent area moment of inertia of the composite section, *θ* is the electromechanical coupling coefficient and *δ*(*x*) is the Dirac delta function. According to the proportional damping criterion, the strain-rate damping coefficient *c_s_* is proportional to the beam stiffness *Y*, and the viscous air damping coefficient *c_a_* is proportional to the mass per unit length of the beam *m*. This allows expressing the displacement *w*(*x,t*) as the summation of modes in the beam at temporal coordinate that provides a convenient approach to obtain the solution from partial derivative equations (PDEs).

(4) $$\begin{array}{l} YI\frac{\partial^{4}w(x,t)}{\partial x^{4}}+c_{s}I\frac{\partial^{5}w(x,t)}{\partial x^{4}\partial t}+c_{a}\frac{\partial w(x,t)}{\partial t}+m\frac{\partial^{2}w(x,t)}{\partial t^{2}}+\vartheta v_{out}(t)\times \left(\frac{d\delta (x)}{dx}-\frac{d\delta (x-L)}{dx}\right)\\ =F_{impat}(t)\delta (x-(L-l_{0})) \end{array}$$

By using the modal expansion method, the displacement *w*(*x,t*) can be represented as an absolutely and uniformly convergent series of the eigenfunctions written in Equation (5) where *φ_r_*(*x*) and *η_r_*(*t*) are the mass normalized eigenfunction and the modal coordinate for the *r*th mode, respectively. *φ_r_*(*x*) is given by Equation (6) where *σ_r_* = (sinh *λ_r_* − sin *λ_r_*)/(cosh *λ_r_* + cos *λ_r_*), and *C_r_* is a modal amplitude constant which can be evaluated by normalizing the eigenfunctions according to the orthogonality conditions. The parameters *λ_r_* are the dimensionless frequency numbers obtained from the frequency function shown in Equation (7) for the cantilever configuration. The undamped natural frequency *ω_r_* can be obtained by Equation (8).

(5) $$w(x,t)=\sum_{r=1}^{\infty }\phi_{r}(x)\eta_{r}(t),$$

(6) $$\phi_{r}(x)=C_{r}\left(\cosh\frac{\lambda_{r}}{L}x-\cos\frac{\lambda_{r}}{L}x-\sigma_{r}(\sinh\frac{\lambda_{r}}{L}x-\sin\frac{\lambda_{r}}{L}x)\right),$$

(7) 1+cosλcoshλ=0,

(8) $$\omega_{r}=\lambda_{r}{}^{2}\sqrt{\frac{YI}{mL^{4}}}.$$

The mechanical equation of motion in modal coordinates can be expressed in Equation (9) where *χ_r_* is the backward coupling term given by Equation (10). It can be seen that *χ_r_* is related to the electromechanical coupling coefficient *θ* which can be expressed in Equation (11). Herein, *Y_p_* and *h_p_* is the Young’s modulus and thickness of the PVDF layer respectively, *h_c_* is the distance of the top of the PVDF layer from the neutral axis and *d*_31_ the piezoelectric constant. The damping term *ζ_r_* is expressed by Equation (12) where both *c_s_* and *c_a_* can be determined from experiments.

(9) $$\frac{d^{2}\eta_{r}(t)}{dt^{2}}+2\zeta_{r}\omega_{r}\frac{d\eta_{r}(t)}{dt}+\omega_{r}{}^{2}\eta_{r}(t)+\chi_{r}v_{out}(t)=F_{impact}(t)\delta (x-(L-l_{0})),$$

(10) $$\chi_{r}=\theta \frac{d\phi_{r}(L)}{dx},$$

(11) $$\theta =-\frac{Y_{p}d_{31}b}{h_{p}}h_{c}{}^{2},$$

(12) $$\zeta_{r}=\frac{c_{s}\omega_{r}}{Y}+\frac{c_{a}}{2m\omega_{r}}.$$

The modal force on the right-hand side of Equation (9) is the average force [35] applied on the beam given by the droplet. It can be written as in Equation (13) where τ* is the corrected impact duration determined following experiments. To guarantee the survival of *F_impact_* in the differential equation of motion, heaviside functions *H*(*t*) are associated. Considering the substrate flexibility and based on typical impact duration [36] defined as *τ* = *D_d_/V_d_*, the corrected impact duration τ* can be written in form of Equation (14). In this expression, the exponent *κ* (>1) depends only on droplet diameter and can be presented by the form of *α·D_d_^β^* + *γ* where the coefficients *α*, *β* and *γ* are determined by experiments as *α* = −0.025, *β* = −0.50, *γ* = 1.37. It can be seen from Equation (14) that for flexible substrate, the droplet diameter *D_d_* and the impact velocity *V_d_* affect the impact duration oppositely that the greater the *D_d_* and the smaller the *V_d_*, the greater the τ* is, and vice versa.

(13) $$F_{impact}(t)=\frac{m_{d}V_{d}^{2}}{D_{d}}(H(t)-H(t-\tau^{*})),$$

(14) $$\tau^{*}=\frac{D_{d}^{\kappa }}{V_{d}^{1/\kappa }}.$$

**Figure 2:**
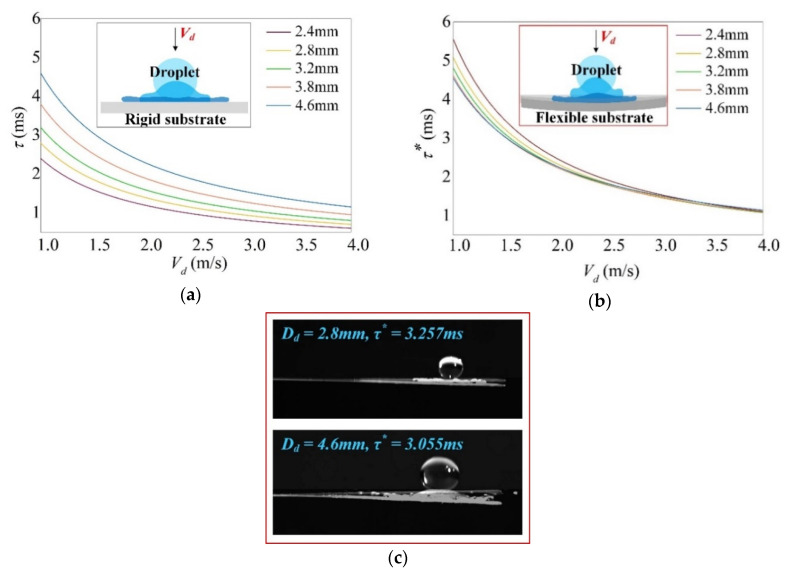
Impact duration of various droplet diameters *D_d_* (i.e., 2.4mm~4.6mm) as a function of impact velocity *V_d_* with (**a**) typical impact duration *τ = D_d_/V_d_* on rigid substrate; (**b**) corrected impact duration τ* (Equation (14)) on flexible substrate and (**c**) comparison of τ* for droplet *D_d_* = 2.8 mm and *D_d_* = 4.6 mm, showing the effect of substrate flexibility.

Figure 2 shows the impact duration for various diameters of droplet on both rigid and flexible substrates. For rigid substrate (Figure 2a), *τ* is much shorter and greatly depends on droplet diameter; whereas for flexible substrate (Figure 2b), all droplet diameters lead to about the same variation of τ* especially for higher impact velocity. It shows that the substrate flexibility has a cushion effect on resisting the impact momentum of droplet which leads to a less droplet spreading. As a result, it narrows the discrepancy of τ* between droplets. As shown in Figure 2c, when water droplets of *D_d_* = 2.8 mm and *D_d_* = 4.6 mm impinge the cantilever beam at the same impact velocity *V_d_* = 1.5 m/s, the τ* is calculated to be 3.257 ms and 3.055 ms respectively. Snapshot for each droplet shows crashing time between instant *t* = 0 ms and *t* = τ*. The τ* of *D_d_* = 4.6 mm is slightly smaller than that of *D_d_* = 2.8 mm. It is associated with the greater droplet inertia of *D_d_* = 4.6 mm which leads to a larger deflection of beam than *D_d_* = 2.8 mm against the same substrate flexibility. The corrected impact duration is shown to be effective in predicting the dynamic outputs of PVDF cantilever under any impact parameters such as the droplet diameter, impact velocity and also the wettability of beam surface.

Considering the electromechanical coupling in 3-1 mode, the constitutive relations of cantilever beam can be expressed in Equation (15) where $s_{11}^{E}$ is the elastic compliance at constant electric field and $\varepsilon_{33}^{T}$ the permittivity at constant stress. For an Euler-Bernoulli beam, the axial strain *S*_1_ is assumed to be proportional to the curvature of the beam at position x and at a certain level y from the neutral axis written in form of Equation (16). The axial stress *T*_1_ can be determined from Hook’s law then the electric field *E*_3_ and the electric displacement *D*_3_ can be obtained.

(15) $$\begin{array}{l} S_{1}=s_{11}^{E}T_{1}+d_{31}E_{3}\\ D_{3}=d_{31}T_{1}+\varepsilon_{33}^{T}E_{3} \end{array}$$

(16) $$S_{1}(x,y,t)=-y\frac{\partial^{2}w(x,t)}{\partial x^{2}}.$$

In this way, expressing the electric field by voltage across the PVDF layer (*E*_3_* = −v_out_*(*t*)*/h_p_*) yields the electrical circuit equation with mechanical coupling shown in Equation (17). The forward coupling term *φ_r_* is expressed in Equation (18) where *h_pc_* is the distance from the center of the PVDF layer to the neutral axis, and $\varepsilon_{33}^{S}$ is the permittivity at constant strain ($\varepsilon_{33}^{S}$ *=* $\varepsilon_{33}^{T}$ − *d*_31_^2^*Y_p_*). Correspondingly, the internal capacitance *C_p_* can be written as in Equation (19). On the other hand, the same governing equation can be obtained by applying the Kirchhoff laws on the RC electrical circuit shown in Figure 1b. Such that the current source term *i*(*t*) can be easily identified (the right-hand side of Equation (17)). In an open-circuit condition, no external electric field is applied on the beam (*E*_3_ *=* 0) and the term *v_out_*(*t*)*/R_load_* tends to zero.

(17) $$\frac{dv_{out}(t)}{dt}+\frac{v_{out}(t)}{R_{load}C_{p}}=\sum_{r=1}^{\infty }\varphi_{r}\frac{d\eta_{r}(t)}{dt},$$

(18) $$\varphi_{r}=-\frac{d_{31}Y_{p}h_{pc}h_{p}}{\varepsilon_{33}^{S}L}\int_{x=0}^{L}\frac{d^{2}\phi_{r}(x)}{dx^{2}}dx=-\frac{d_{31}Y_{p}h_{pc}h_{p}}{\varepsilon_{33}^{S}L}{\frac{d\phi_{r}(x)}{dx}|}_{x=L},$$

(19) $$C_{p}=\frac{\varepsilon_{33}^{S}bL}{h_{p}}.$$

Overall, Equations (9) and (17) are the basic electromechanical equations of a distributed parameter model for the PVDF cantilever used in the study. Considering the first three modes of vibration, the simulated results obtained by the multi-mode model developed are compared and validated by in-laboratory impact experiments. Based on these results, the dynamic response of the PVDF cantilever excited by impacting droplets can be systematically studied whereby the impact mechanisms upon droplet-substrate interactions can be further complemented.

## 3. Experiment Setup

**Figure 3:**
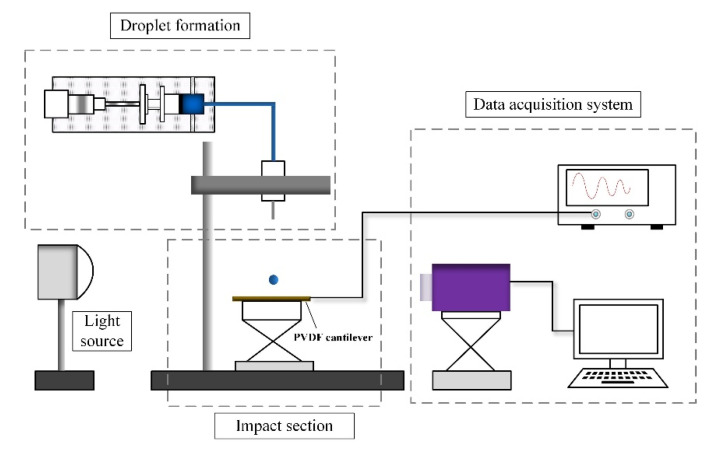
Experiment setup for impact tests.

To produce artificial raindrops, water droplets are formed from blunt needles (Type of 10G, 15G, 19G, 21G, 25G) which is supplied by a micropump, shown as in Figure 3. Releasing water droplet at different heights allows to obtain various impact velocities. The practical impact velocity is measured from captured images obtained by a high-speed camera (NAC Company, HX-7 s, Tokyo, Japan) equipped with a light source (200W direct light). The droplet diameter is also measured from captured images just after it is squeezed from the needle. The PVDF cantilever sensor is fixed at the workbench and connected to a digital oscilloscope. In an open-circuit condition, the PVDF sensor is connected with a pure resistance of 100 MΩ and the cut-off frequency of oscilloscope is 0.145 Hz which allows the acquisition of low frequency electric signals. The data acquisition system shown in Figure 3 is used to capture the droplet dynamics occurred on beam surface and acquire the electric output simultaneously.

Untreated PVDF sensor processes a hydrophilic surface. To prepare a SH beam surface, the upper surface of the sensor is coated with nanoparticles by using commercially available spray (Rust-Oleum NeverWet). To access the wettability, the apparent advancing and receding contact angles (i.e., θ_a_* and θ_r_*) are measured by the stationary droplet method for both surfaces. The measured values for the SH surface are θ_a_* = 155° ± 1.2° and θ_r_*=148° ± 2.0°, and those for the hydrophilic surface are θ_a_* = 91.3° ± 3.2° and θ_r_* = 49° ± 2.6°. When water droplets (2.4 mm to 4.6 mm) are released and impinge the cantilever tip, to generate the greatest voltage output as well to avoid the droplet slipping off the surface earlier, the impact location is selected at a distance of *l*_0_ = 7.0 mm from the beam edge to ensure the maximum spreading of the largest droplet. Each impact test is repeated three times to guarantee the reproducibility and precision of experiments.

## 4. Discussion

### 4.1. Impact on a Hydrophilic Beam

**Figure 4:**
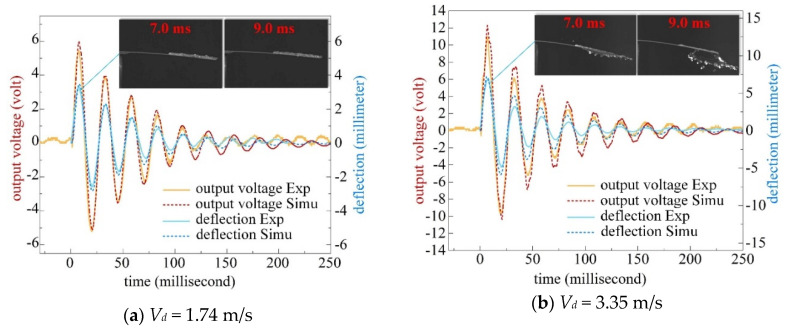
Comparison of cantilever tip deflection and output voltage between experiments and simulations for impact velocity of (**a**) *V_d_* = 1.74 m/s and (**b**) *V_d_* = 3.35 m/s.

Figure 4 shows the dynamic outputs as a water droplet *D_d_* = 4.6 mm impacts on the untreated beam surface (i.e., hydrophilic surface) under open-circuit condition. Both the deflection of the cantilever tip *w*(*L*,*t*) and the output voltage *v_out_*(*t*) are compared between experimental and simulated results. It can be seen that the voltage follows the beam deflection in time. For lower impact velocity (Figure 4a), the simulated results agree well with the experiments. For the higher impact velocity (Figure 4b), higher order modes of vibration take effect especially around peak values of voltage. Besides, a fraction of water droplet spills off the beam from both side edges after the maximum droplet spreading, as depicted in the insets of Figure 4b. As a result, the simulated voltage is generally higher than experiments in addition to the peak voltage which is less affected by the spill of water. Indeed, the water spilling phenomenon can lead to mechanical loss of the system and occurs with higher impact velocities. It depends on the beam dimensions and droplet spreading diameter. Nevertheless, the multi-mode model can still predict the value of peak voltage but will overestimate the total energy harvested from a hydrophilic beam surface.

### 4.2. Droplet-Substrate Interaction Mechanisms

**Figure 5:**
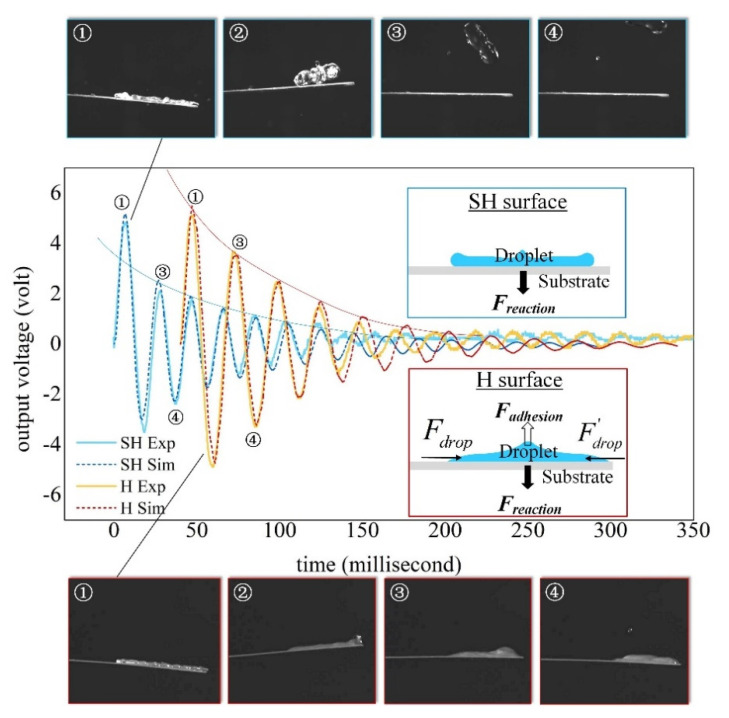
Dynamic response (voltage comparison between experiments and simulations with snapshots showing droplet dynamic behaviors at typical instants) as a droplet of *D_d_* = 4.6 mm and *V_d_* = 1.5 m/s impinge both the SH and H surfaces.

Compared to untreated beam surfaces (i.e., hydrophilic surfaces), the mechanisms of droplet-substrate interactions of SH beam surfaces are changed. Figure 5 compares the output voltage from SH and H surfaces for a droplet *D_d_* = 4.6 mm with an impact velocity *V_d_* = 1.5 m/s. Snapshots on top (SH surface) and bottom (H surface) of the figure are related to droplet dynamics captured at typical instants. For both surfaces, the cantilever undergoes upward motion from ① to ② during which the droplet adheres on the H surface while the droplet recoils and lifts up from the SH surface. After ②, the H cantilever vibrates together with the total droplet, whereas the SH cantilever undergoes free vibration.

As shown in the insets of Figure 5 concerning the stage of ① to ②, forces applied on the cantilever beam are different when surface wettability changes. For the H surface, there exists an adhesion force *F_adhsion_* given by the droplet which promotes the upward motion of beam. The droplet also gives a reaction force *F_reaction_* to the beam in the opposite direction which hinders the beam from going upward. Moreover, the spreading and oscillation of the droplet over the beam can generate horizontal forces (*F_drop_* and *F^’^_drop_*) which has negligible effect on the upward motion of beam. It can be seen that the simulated results for H surface fit well with the experiments. However, in the modeling procedure, no other than the impact force of droplet is applied as the modal force. It indicates that the couple of force *F_adhsion_* and *F_reaction_* are somewhat compensated between each other during the deformation of the beam.

To model SH surfaces without considering the adhesion force, the reaction force *F_reaction_* can be applied as an additional modal force during the period *π*/2*ω*_0_~3*π*/2*ω*_0_ (i.e., ①–②) with *ω*_0_ the first-mode undamped frequency. The magnitude of *F_reaction_* depends on droplet inertia. Larger droplet with higher impact velocity can generate the greater reaction force where the dimensionless numbers *We* and *Re* related to droplet inertia should be involved. Here, we express the reaction force as a ratio of the impact force, an empirical expression of *F_reaction_* is written in Equation (20) where D* (=2.3 mm) is a reference droplet diameter. Therefore, the mechanical equation of motion in modal coordinates for SH cantilever beam becomes Equation (21).

(20) Freaction(t)=6.6⋅(DdD*)2.1⋅We−1⋅Re−0.02⋅Fimpact⋅(H(t)−H(t−1.5πω0)),

(21) d2ηr(t)dt2+2ζrωrdηr(t)dt+ωr2ηr(t)+χrvout(t)=Fimpat(t)δ(x−(L−l0))+6.6⋅(DdD*)2.1⋅We−1⋅Re−0.02⋅Fimpact⋅(H(t)−H(t−1.5πω0))

By applying the reaction force in the model, the simulated results agree well with the experiments shown as in Figure 5. Note that, the second peak value of voltage (i.e., ③) of a SH surface is much lower than that of an H surface. Actually, a damped harmonic vibration of SH cantilever can only be achieved after the vanishing of the reaction force. The damping ratio *ζ_r_* of the first-mode vibration is shown to be 0.047 and 0.065 for the SH and H surfaces respectively. Therefore, the SH surface seems to be destructive in generating higher voltage output and constructive in conserving the mechanical energy from viscous damping.

### 4.3. Impact on a Super-Hydrophobic Beam

#### 4.3.1. Regime Transition of Droplet Impact Mechanism

**Figure 6:**
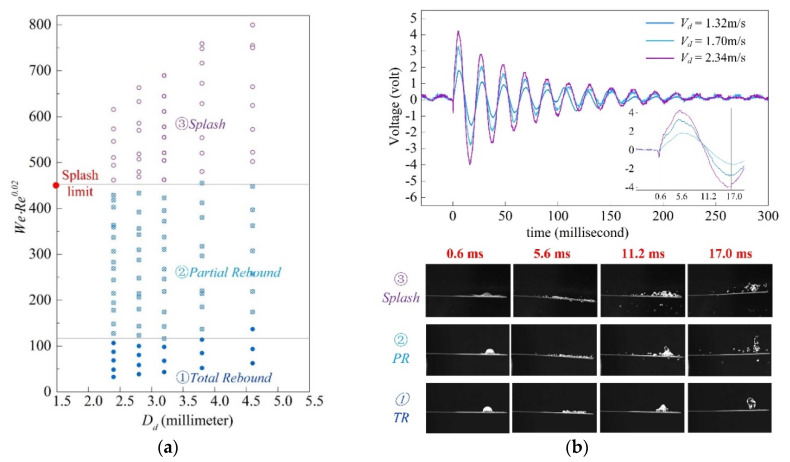
Typical regimes of droplet determined by the value of term ‘*We·Re*^0.02^*′* obtained from experiments: (**a**) regimes of ‘Total Rebound’, ‘Partial Rebound’ and ‘Splash’ are presented by different colors of points, and the transition limit can be approximately determined by specific values of ‘*We·Re*^0.02^*′*; (**b**) examples of voltage outputs for a droplet *D_d_* = 2.8 mm experiencing regime transitions as the impact velocity *V_d_* varies from 1.32 m/s to 2.34 m/s.

According to experimental results of SH surfaces (Figure 6a), three typical regimes of droplet (i.e., “*Total Rebound*” (*TR*), “*Partial Rebound*” (*PR*) and “*Splash*”) are determined by the value of *WeRe*^0.02^ for all droplet diameters. Noted that the term *We*^−1^*Re*^−0.02^ also exists in Equation (20) which shows the interplay between droplet dynamics and the impact force applied on the beam. Because of the substrate flexibility, the dependence of the impact regime on *Re* begins to weaken compared to the splash parameter shown in Equation (3). As shown on top of the Figure 6b, the voltage output of three impact tests for each regime is presented using a droplet of *D_d_* = 2.8 mm with impact velocity *V_d_* varying from 1.32 m/s to 2.34 m/s. Snapshots on bottom of Figure 6b show the relative instants indicated above. For regime of *TR*, the surface tension force maintains the droplet as a whole till it leaving from the surface. Besides, we distinguish the regime *Splash* from the regime *PR* by the generation of satellite drops at the initial stage of interactions shown as in test ③. As a result, the droplet in *Splash* regime can lead to severe kinetic energy loss delivered by these satellite drops.

#### 4.3.2. Effect of Droplet Splash

**Figure 7:**
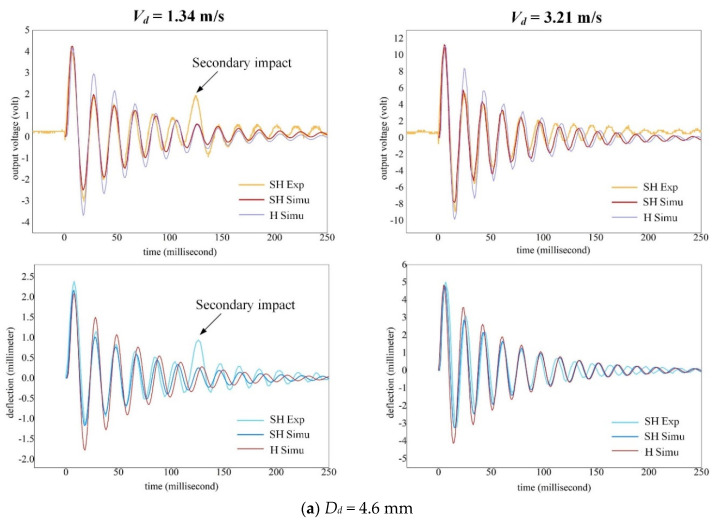

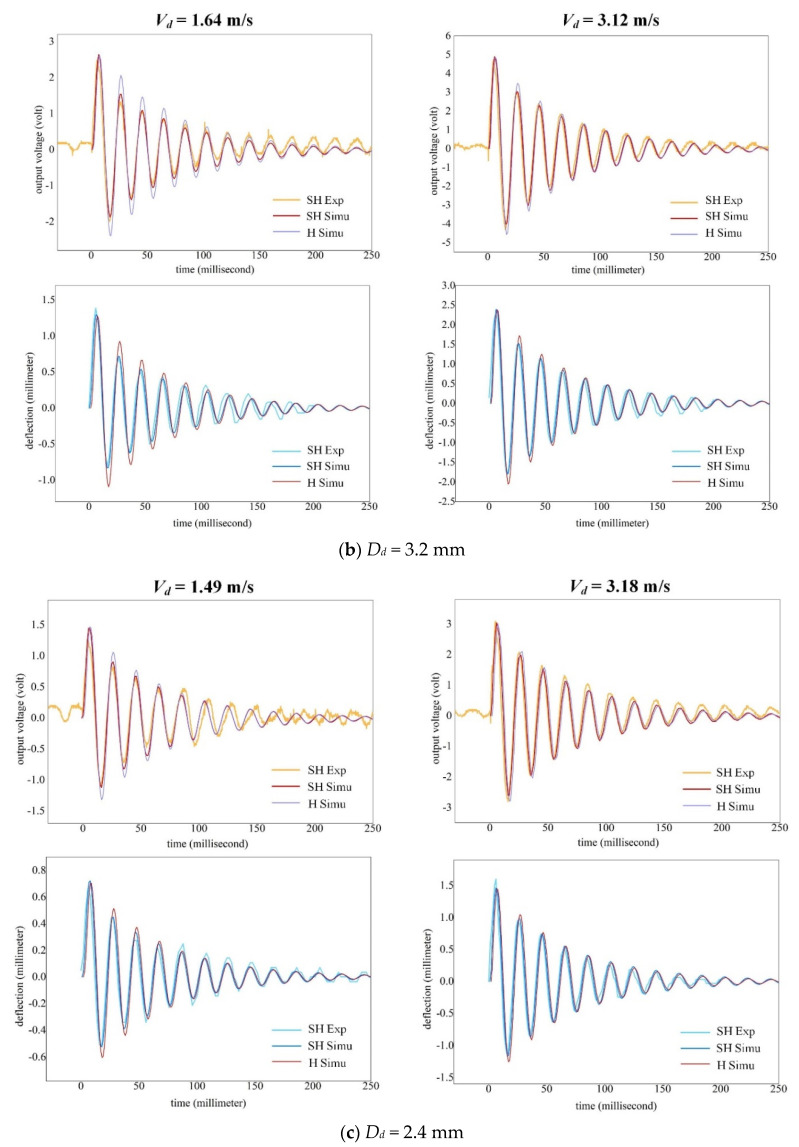
Comparison of the output voltage *v_out_*(*t*) and deflection *w*(*L,t*) for both SH and H surfaces under excitation of various droplets: (**a**) *D_d_* = 4.6 mm; (**b**) *D_d_* = 4.6 mm; (**c**) *D_d_* = 2.4 mm.

To study the effect of droplet splash on dynamic response of the cantilever, the voltage across the PVDF layer *v_out_*(*t*) and the deflection of cantilever tip *w*(*L,t*) are analyzed by varying impact parameters. As the reaction force is a function of droplet diameter (Equation (20)), Figure 7 illustrates the *v_out_*(*t*) and *w*(*L,t*) for various droplet diameters when they transit into *Splash* regime. As shown in Figure 7a, the simulated results of a SH surface fit well with the experiments for the droplet *D_d_* = 4.6 mm apart from the secondary impact occurred in experiments. It concerns the lifted droplet re-impinges the beam surface in *TR* regime (i.e., *V_d_* = 1.34 m/s). This phenomenon cannot be predicted by the model because the present model only considers single droplet impact. It can also be seen that the simulated results for an H surface are generally higher than that of a SH surface. When the droplet transits into *Splash* regime (i.e., *V_d_* = 3.21 m/s), similar results can be obtained except that the relative difference of *v_out_*(*t*) and *w*(*L,t*) between two surfaces are slightly lower than the former.

For smaller droplets as shown in Figure 7b,c, the difference of *v_out_*(*t*) and *w*(*L,t*) between two surfaces also becomes smaller especially for droplets in *Splash* regime (i.e., *V_d_* = 3.12 m/s for droplet *D_d_* = 3.2 mm and *V_d_* = 3.18 m/s for droplet *D_d_* = 2.4 mm). It indicates that the smaller the droplet diameter, the smaller the difference of *v_out_*(*t*) and *w*(*L,t*) between two surfaces is. Viewed from another perspective, the reaction force becomes inefficient when droplets transit into *Splash* regime for small-scaled droplets such as *D_d_* ≤ 2.4 mm. In this case, the SH surface is able to generate the same voltage output as the H surface. Note that, as previously demonstrated in Section 4.1, an H surface can trigger the water spilling phenomenon in high regime of impact velocities, leading to a decreased voltage output. By comparison, no water can be left on a SH surface, thus the voltage output can be accurately predicted with less effect of other parameters such as the beam dimension. Therefore, in *Splash* regime of droplet, a SH surface can generate even higher voltage output than an H surface under the same impact parameters. Developing the expression of the reaction force shown in Equation (20) leads to the relationship written in Equation (22).

(22) $$F_{reaction}=0.02643\cdot D_{d}^{3.48}$$

**Figure 8:**
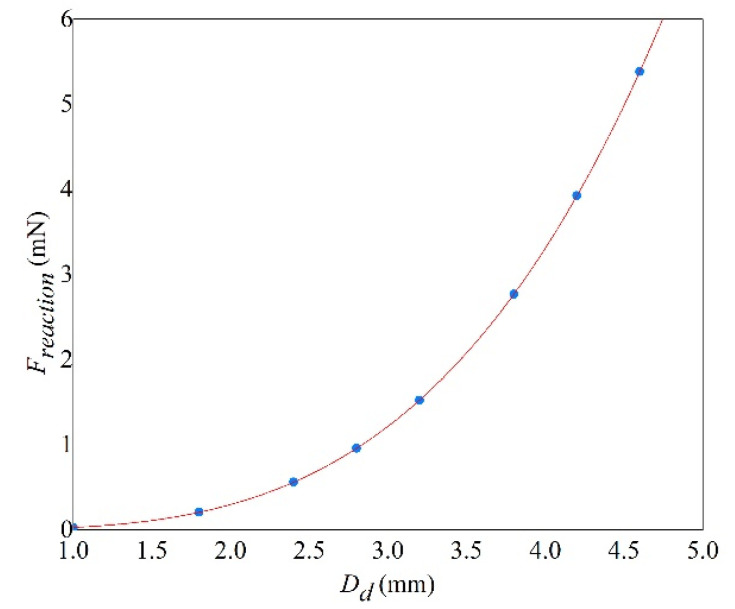
The reaction force as a function of droplet diameter.

Figure 8 shows the variation of *F_reaction_* as a function of the droplet size *D_d_*. It shows that *F_reaction_* increases monotonously with the droplet diameter. For a given droplet diameter, the reaction force is constant. Overall, the multi-mode model is proved to be flexible and robust in predicting the electric output and mechanical motion of cantilever beam for both SH and H surfaces whereby the droplet splash is shown to have a positive effect on dynamic output.

#### 4.3.3. Dynamic Response under Excitation of Raindrops

In this section, the model is applied to real rain conditions where the terminal velocity of raindrops is adopted as the input parameters. It can be estimated [37] as a function of the released height *H* given by Equation (23) where *A* = 3*c_f_ρ_air_*/(4*ρ_d_D_d_*) with *ρ_air_* and *ρ_d_* the density of air and raindrops respectively, *D_d_* is the raindrop diameter and *c_f_* the friction coefficient (= 0.796).

(23) $$V_{T}(H)=\sqrt{\frac{g}{A}\left(1-e^{-2AH}\right)}$$

**Figure 9:**
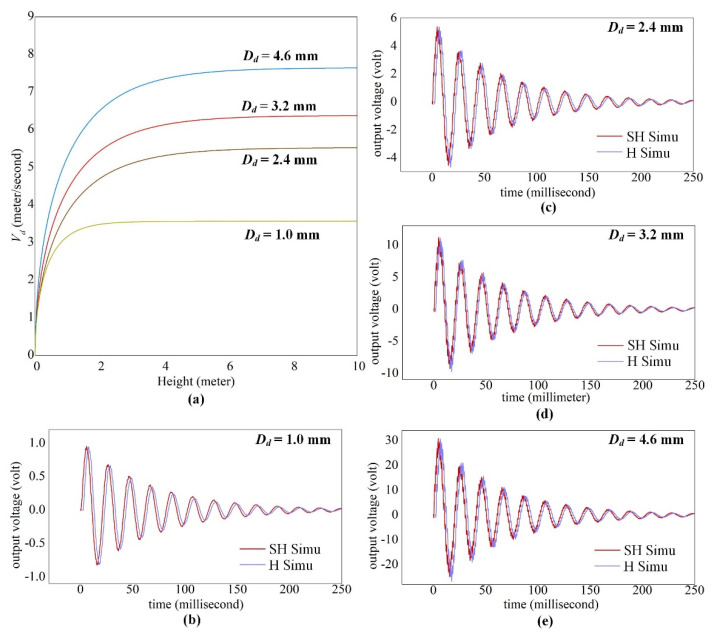
Voltage output under terminal velocity of raindrops with (**a**) the terminal velocity estimation; and the simulated voltage comparison between SH and H surfaces for droplet diameters of (**b**) *D_d_* = 1.0 mm, (**c**) *D_d_* = 2.4 mm, (**d**) *D_d_* = 3.2 mm and (**e**) *D_d_* = 4.6 mm.

Figure 9a illustrates the velocity evolution for a wide range of droplet diameters. By applying the terminal velocity *V_T_*, the simulated voltage from both SH and H beam surfaces are shown in Figure 9b–e. When the *V_T_* is achieved, all droplets are supposed to transit into *Splash* regime and little difference of voltage between surfaces are obtained even for larger droplets (i.e., *D_d_* > 2.4 mm). However, the small-scaled droplet of *D_d_* = 1.0 mm enables the cantilever to undergo first-mode dominated vibration, whereas larger droplets can excite higher order modes of vibration for the cantilever. Therefore, it is necessary to consider the multi-mode effect on cantilever sensor subjected to droplet impact. Considering the water spilling effect on the H surface again, the voltage generated from the SH surface should be practically greater than or equal to that from the H surface. Hence, the SH surfaces is more advantageous in electrical energy collection than the hydrophilic sensor surfaces used before.

### 4.4. Energy Collected from the SH and H Beams

**Figure 10:**
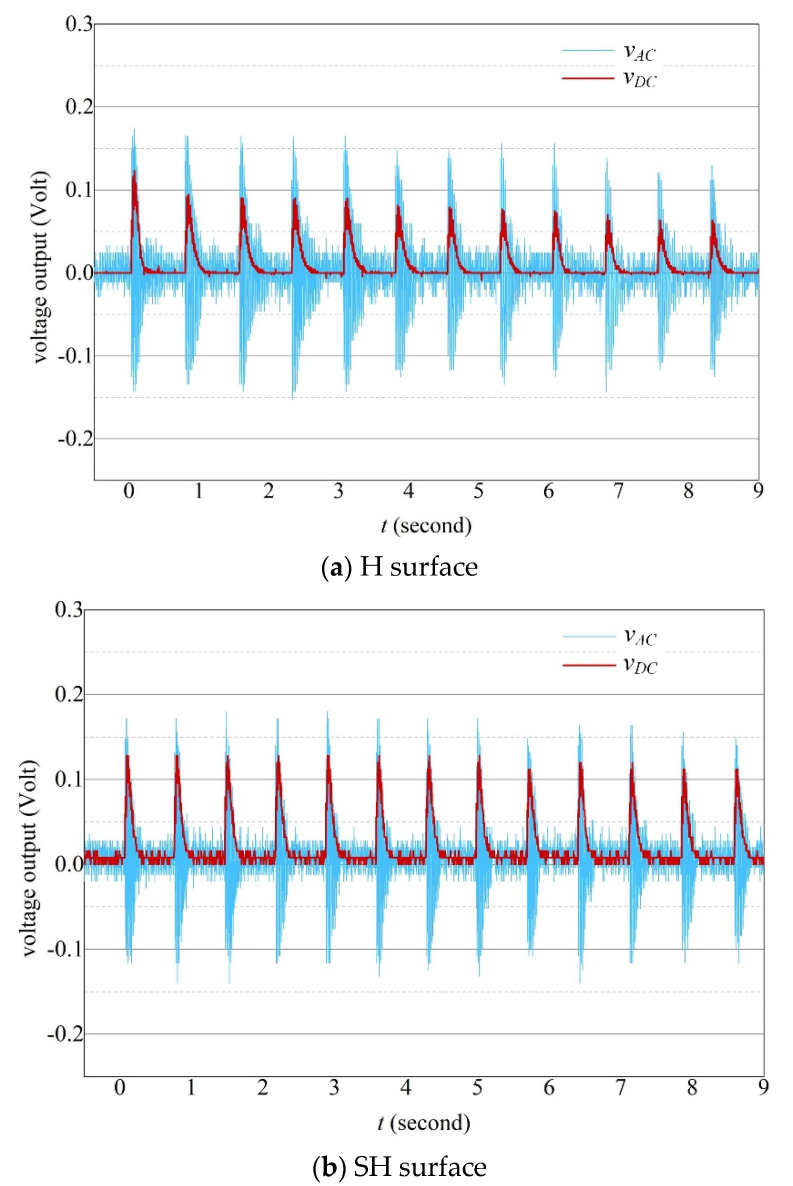
Closed-circuit voltage under successive impacts of a droplet *D_d_* = 3.2 mm from both the (**a**) H surface and the (**b**) SH surface.

To enhance the performance of SH surfaces in rain energy harvesting, a trial of tests is conducted considering successive impacts of droplets as in the real rain condition. Figure 10 compares the closed-circuit voltage measured from both SH and H beam surfaces with an external load *R_load_* = 470 kΩ. A standard bridge rectifying circuit is used and both of the DC voltage *v_DC_*(*t*) and the AC voltage *v_AC_*(*t*) are presented. The total electrical energy collected can be obtained by integrating the *v_DC_*(*t*) over a time period *t_total_* given by Equation (24). It is calculated for the first 12 water droplets.

(24) $$E=\frac{1}{R_{load}}\int_{0}^{t_{total}}v_{DC}{\left(t\right)}^{2}dt$$

It can be seen (Figure 10a) that the peak voltage of *v_DC_*(*t*) for a H surface decreases gradually as the number of accumulated droplets increases. It is associated with the water layer accumulated on the beam surface which hinders the deformation of the cantilever beam. The total energy collected is calculated to be 8nJ. Although it was stated [31] that the water ripples can cause additional gravity loads on the beam surface which leads to greater voltage output, our results show that the depth of water layer in our study is not sufficient which is restricted by the beam dimension. Thus, the presence of water layer finally has a negative effect on voltage generation. By comparison (Figure 10b), the SH surface generates constant peak voltage of *v_DC_*(*t*) with a total energy of 28.5 nJ. Overall, the SH surface performs better and shows itself as a good candidate in rain energy harvesting applications. However, the performance of the super-hydrophobic PVDF is also affected by the durability of the super-hydrophobic surface, and the actual design should consider the failure of the super-hydrophobic coating.

## 5. Conclusions

A novel multi-mode model for polyvinylidene fluoride cantilever beams is developed and validated by impact experiments. In the model, the corrected impact duration determined from experiments allows to estimate the crashing time of droplet on flexible substrate for various impact parameters (e.g., droplet diameter, impact velocity and etc.). The modeling of the reaction force between droplet and substrate facilitates to study the effect of surface wettability on dynamic outputs, which is also related to regime transition of the droplet. The droplet splash is shown to have a positive effect on electric output. The splash limit is experimentally determined and greatly dominated by the Weber number (We) on flexible substrates. For a hydrophilic (H) beam surface, the water spilling phenomenon leads to a decrease of voltage output but has little influence on peak values of the outputs. For a super-hydrophobic (SH) surface, predicted results were in good agreement with the experiments. Both the output voltage and cantilever deflection can be accurately predicted whatever the regime of droplet. The SH treatment of beam surface provides an insight into understanding the electromechanical behaviors of energy harvester in a quantitative way. The performance of SH surface is proved to be superior to hydrophilic surface for the following reasons: first, it shows a great potential in generating higher voltage than the H surface especially in Splash regime of small-scaled droplets under single droplet impact condition; second, the SH surface can generate constant peak voltage and higher energy collected compared to the H surface which is affected by the accumulated water layer over the beam surface under successive droplet impacts condition; third, SH surface shows the possibility of estimating the droplet size so as the rain intensity because the voltage output is greatly dominated by the short duration of droplet-substrate interactions and is less affected by other parameters such as the beam dimension. Besides, the SH surface is specifically advantageous in self-cleaning or anti-freezing utilizations where a water repellent surface is needed. Overall, the utilization of SH beam surface is encouraged in improving the impact efficiency in rain energy harvesting applications. Moreover, results of the study may also be applied in other practical utilizations related to droplet impacting on flexible substrate.

## References

[1] Guigon R., Chaillout J.-J., Jager T., Despesse G. (2008). Harvesting raindrop energy: Experimental study. Smart Mater. Struct..

[2] Guigon R., Chaillout J.-J., Jager T., Despesse G. (2008). Harvesting raindrop energy: Theory. Smart Mater. Struct..

[3] Viola F., Romano P., Miceli R., Acciari G., Spataro C. (2014). Piezoelectric model of rainfall energy harvester. Proceedings of the 2014 Ninth International Conference on Ecological Vehicles and Renewable Energies (EVER).

[4] Helseth L., Wen H. (2017). Evaluation of the energy generation potential of rain cells. Energy.

[5] Fabio V. (2018). Comparison among different rainfall energy harvesting structures. Appl. Sci..

[6] Doria A., Fanti G., Filipi G., Moro F. (2019). Development of a Novel Piezoelectric Harvester Excited by Raindrops. Sensors.

[7] Ong Z.-Z., Wong V.-K., Ho J.-H. (2016). Performance enhancement of a piezoelectric rain energy harvester. Sensors Actuators A Phys..

[8] Ilyas M.A., Swingler J. (2017). Towards a prototype module for piezoelectric energy harvesting from raindrop impacts. Energy.

[9] Chua K.G., Hor Y.F., Lim H.C. (2016). Raindrop kinetic energy piezoelectric harvesters and relevant interface circuits: Review, issues and outlooks. Sens. Transducers.

[10] Wong C.H., Dahari Z., Jumali M.H., Mohamed K., Mohamed J.J. (2017). Simulation and Fabrication of Wagon-Wheel-Shaped Piezoelectric Transducer for Raindrop Energy Harvesting Application. J. Electron. Mater..

[11] Chin-Hong W., Dahari Z., Manaf A.A., Sidek O., Miskam M.A., Mohamed J.J., Miskam M.A. (2013). Simulation of Piezoelectric Raindrop Energy Harvester. Proceedings of the IEEE TENCON Spring Conference 2013.

[12] Vatansever D., Hadimani R.L., Shah T., Siores E. (2011). An investigation of energy harvesting from renewable sources with PVDF and PZT. Smart Mater. Struct..

[13] Jellard S., Pu S.-H., Chen S., Yao K., White N.M. (2019). Water droplet impact energy harvesting with P(VDF-TrFE) piezoelectric cantilevers on stainless steel substrates. Smart Mater. Struct..

[14] Patel I., Siores E., Shah T. (2010). Utilisation of smart polymers and ceramic based piezoelectric materials for scavenging wasted energy. Sensors Actuators A Phys..

[15] Ilyas M.A., Swingler J. (2015). Piezoelectric energy harvesting from raindrop impacts. Energy.

[16] Wong C.H., Dahari Z., Manaf A.A., Miskam M.A. (2014). Piezoelectric Beam Length Optimization for Raindrop Energy Harvesting Application. Appl. Mech. Mater..

[17] Izrin I.M., Dahari Z. Power Converter for Raindrop Energy Harvesting Application: Full-Wave Rectifier. Proceedings of the 2017 IEEE 15th Student Conference on Research and Development (SCOReD).

[18] Acciari G., Caruso M., Miceli R., Riggi L., Romano P., Schettino G., Viola F. (2018). Piezoelectric Rainfall Energy Harvester Performance by an Advanced Arduino-Based Measuring System. IEEE Trans. Ind. Appl..

[19] Wong V.-K., Ho J.-H., Sam H.-K. (2016). On accumulation of water droplets in piezoelectric energy harvesting. J. Intell. Mater. Syst. Struct..

[20] Viola F., Romano P., Miceli R., Acciari G. (2013). Harvesting Rainfall Energy by Means of Piezoelectric Transducer. Proceedings of the 2013 International Conference on Clean Electrical Power (ICCEP).

[21] Wong V.-K., Ho J.-H., Yap E.H., Chai A.B. (2017). Dynamics of a piezoelectric energy harvester in a simulated rain environment. Proc. Inst. Mech. Eng. Part C J. Mech. Eng. Sci..

[22] Wong V.-K., Ho J.-H., Chai A.B. (2017). Performance of a piezoelectric energy harvester in actual rain. Energy.

[23] Wong C.H., Dahari Z. (2017). Development of Vibration-Based Piezoelectric Raindrop Energy Harvesting System. J. Electron. Mater..

[24] Biswas P.V., Uddin M.A. Harnessing raindrop energy in Banglagesh. Proceedings of the International Conference on Mechanical Engineering 2009 (ICME2009).

[25] Hassan A., Wong C.H., Dahari Z. (2017). An Analytical Study of Output Voltage Profile Generated from Raindrop Energy.

[26] Perera K.C.R., Sampath B.G., Dassanayake V.P.C. (2014). Harvesting of Kinetic Energy of the Raindrops. Int. J. Energy Power Eng..

[27] Mundo C., Sommerfeld M., Tropea C. (1995). Droplet-wall collisions: Experimental studies of the deformation and breakup process. Int. J. Multiph. Flow.

[28] Roundy S., Wright P.K. (2004). A piezoelectric vibration based generator for wireless electronics. Smart Mater. Struct..

[29] Erturk A., Inman D. (2008). On Mechanical Modeling of Cantilevered Piezoelectric Vibration Energy Harvesters. J. Intell. Mater. Syst. Struct..

[30] Sodano H.A., Park G., Inman D.J. (2004). Estimation of Electric Charge Output for Piezoelectric Energy Harvesting. Strain.

[31] Wong V.-K., Ho J.-H., Yap E. (2014). Dynamics of a piezoelectric beam subjected to water droplet impact with water layer formed on the surface. J. Intell. Mater. Syst. Struct..

[32] Erturk A., Inman D.J. (2008). A Distributed Parameter Electromechanical Model for Cantilevered Piezoelectric Energy Harvesters. J. Vib. Acoust..

[33] Beeby S.P., O’Donnell T.B. (2009). Energy Harvesting Technologies.

[34] Doria A., Fanti G., Moro F. (2019). Response of a Piezoelectric Harvester to Impacts Generated by Rain-Drops. Proceedings of the 2019 Fourteenth International Conference on Ecological Vehicles and Renewable Energies (EVER).

[35] Soto D., De Larivière A.B., Boutillon X., Clanet C., Quéré D. (2014). The force of impacting rain. Soft Matter.

[36] Imeson A., Vis R., De Water E. (1981). The measurement of water-drop impact forces with a piezo-electric transducer. Catena.

[37] Range K., Feuillebois F. (1998). Influence of Surface Roughness on Liquid Drop Impact. J. Colloid Interface Sci..

